# Supplementary material for: The catalytic activity of TCPTP is auto-regulated by its intrinsically disordered tail and activated by Integrin alpha-1
Source: Nat Commun. 2022 Jan 10;13:94. doi: 10.1038/s41467-021-27633-6 (PMC8748766; doi:10.1038/s41467-021-27633-6)
Supplement: Supplementary file 1 — Supplementary Information File [file 41467_2021_27633_MOESM1_ESM.pdf]

Supplementary Information

The catalytic activity of TCPTP is auto-regulated by its intrinsically disordered tail and activated by Integrin alpha-1

Jai Prakash Singh[1,2,3,#], Yang Li[4,#], Yi-Yun Chen[1,5], Shang-Te Danny Hsu[1,2,6], Rebecca Page[7], Wolfgang Peti[4,*] & Tzu-Ching Meng[1,2,6,*]

[1]Institute of Biological Chemistry, Academia Sinica, 128 Academia Road Sec. 2, Nankang, Taipei 115, Taiwan; [2]Chemical Biology and Molecular Biophysics, Taiwan International Graduate Program, Academia Sinica, 128 Academia Road Sec. 2, Nankang, Taipei 115, Taiwan; [3]Department of Chemistry, National Tsing-Hua University, 101 Kuang-Fu Road Sec. 2, Hsinchu 300, Taiwan; [4]Department of Molecular Biology and Biophysics, University of Connecticut Health Center, Farmington, CT 06030, USA; [5]Academia Sinica Common Mass Spectrometry Facilities for Proteomics and Protein Modification Analysis, 128 Academia Road Sec. 2, Nankang, Taipei 115, Taiwan; [6]Institute of Biochemical Sciences, National Taiwan University, 1 Roosevelt Road Sec. 4, Taipei 106, Taiwan; [7]Department of Cell Biology, University of Connecticut Health Center, Farmington, CT 06030, USA.

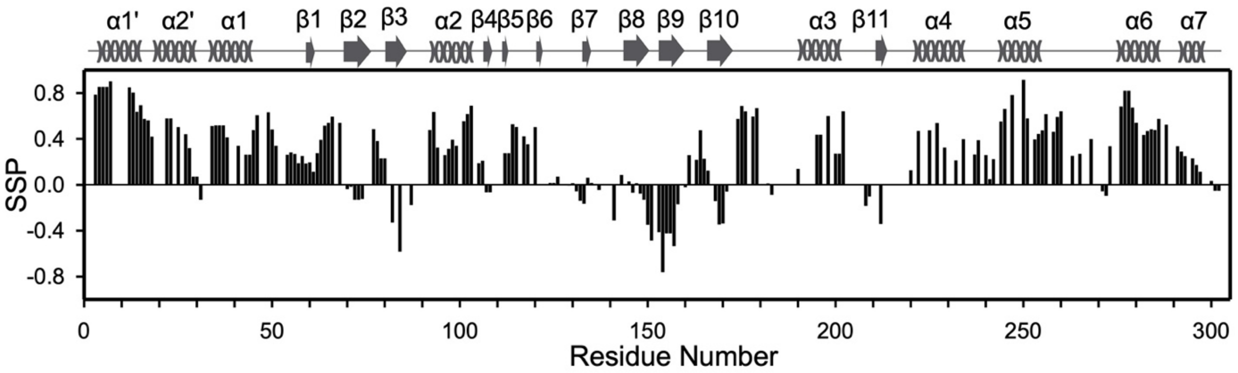

Supplementary Figure 1: $TCPTP_{CAT}$ secondary structure. Secondary-structure propensity (SSP) data plotted vs. $TCPTP_{CAT}$ residue numbers. (SSP > 0, α helix; SSP < 0, β strand). Above: secondary structure from the crystal structure of $TCPTP_{CAT}$ (PDB: 1L8K).

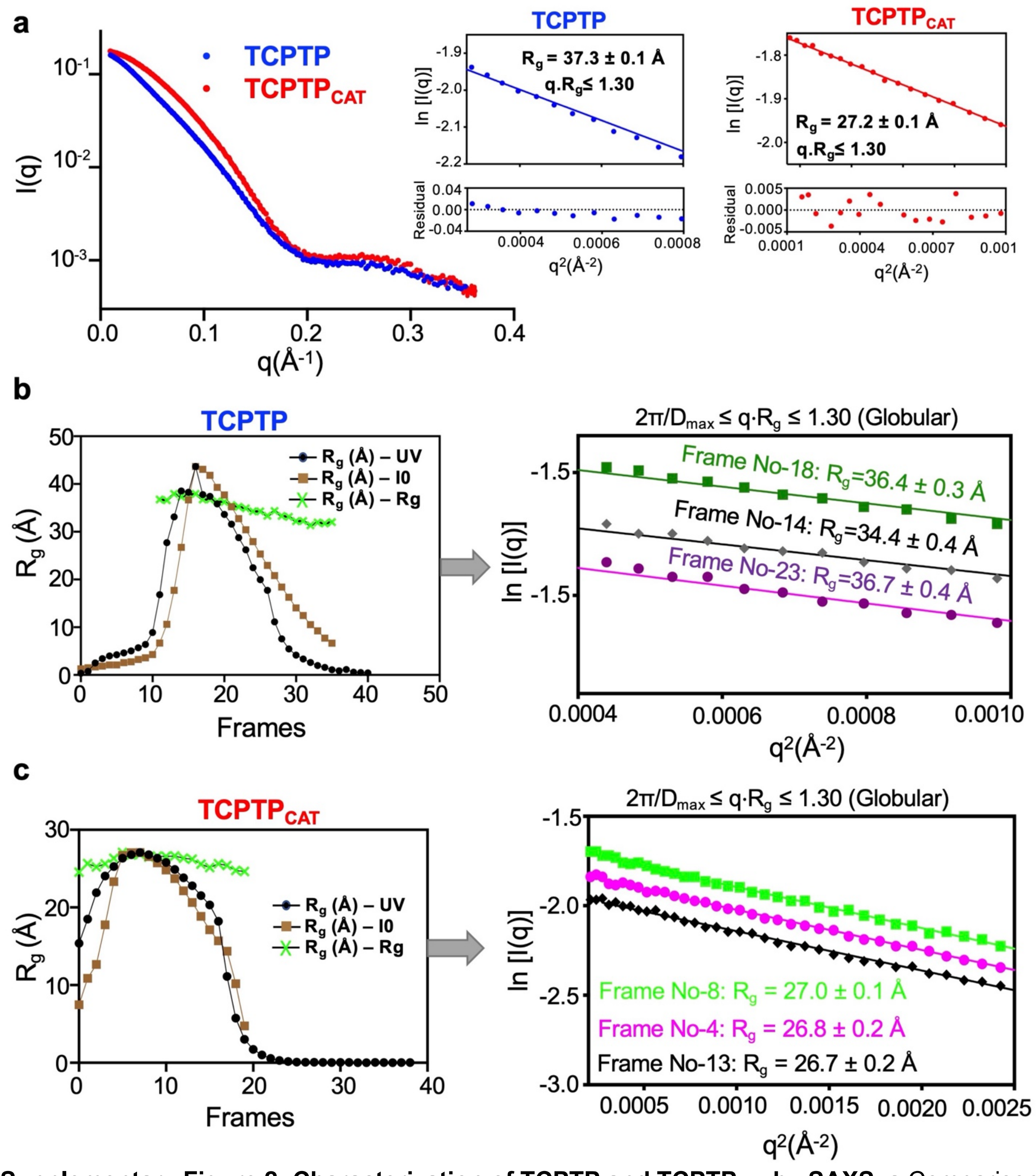

Supplementary Figure 2: Characterization of TCPTP and $TCPTP_{CAT}$ by SAXS. a Comparison of SAXS scattering data and their Guinier plot (q_{max} x R_g<1.3) for TCPTP (blue) and $TCPTP_{CAT}$ (residues 1-314, red). **b** R_g distribution of TCPTP across the total SAXS frames for which data were collected and Guinier plotted (q_{max} x R_g<1.3) from three individual frames to show consistent R_g value. **c** R_g distribution of $TCPTP_{CAT}$ across the total SAXS frames for which data were collected and Guinier plotted (q_{max} x R_g<1.3) from three individual frames to show consistent R_g value. Source data are provided as a Source Data file.

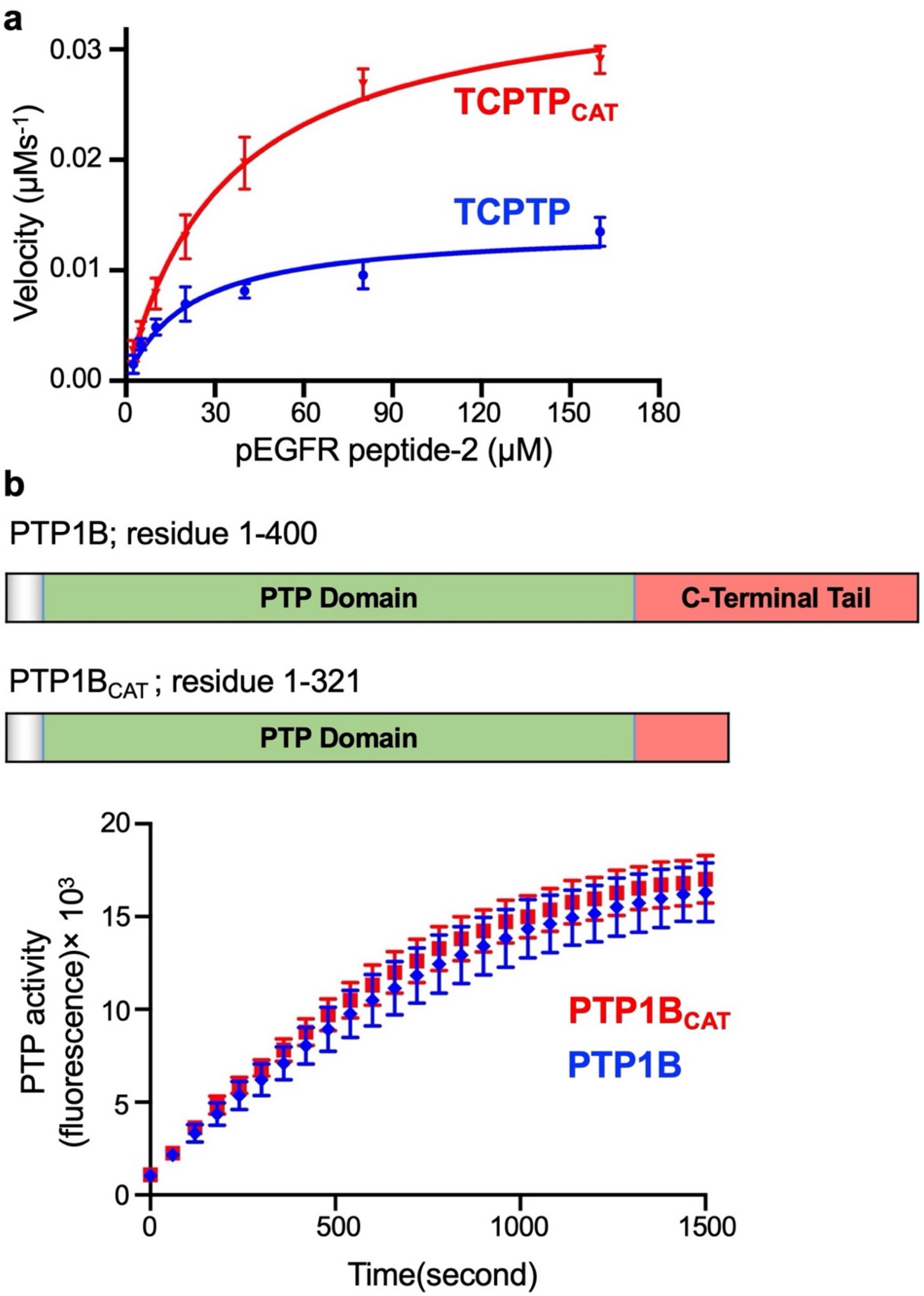

Supplementary Figure 3: Comparison of the catalytic activity for TCPTP and PTP1B. a Catalytic activity of TCPTP and $TCPTP_{CAT}$ (residues 1-314) against pEGFR peptide-2, derived from the EGFR's kinase domain activation loop. Data are presented as the mean of nine independent reactions (*n* = *9*; mean ± SE). **b** Catalytic activity comparison of PTP1B and $PTP1B_{CAT}$ using DiFMUP. Schematic representation of PTP1B (PTP1B) and catalytic domain ($PTP1B_{CAT}$) constructs used in the assay are shown. Data are presented as the mean of six independent reactions (*n* = *6*; mean ± SE). Source data are provided as a Source Data file.

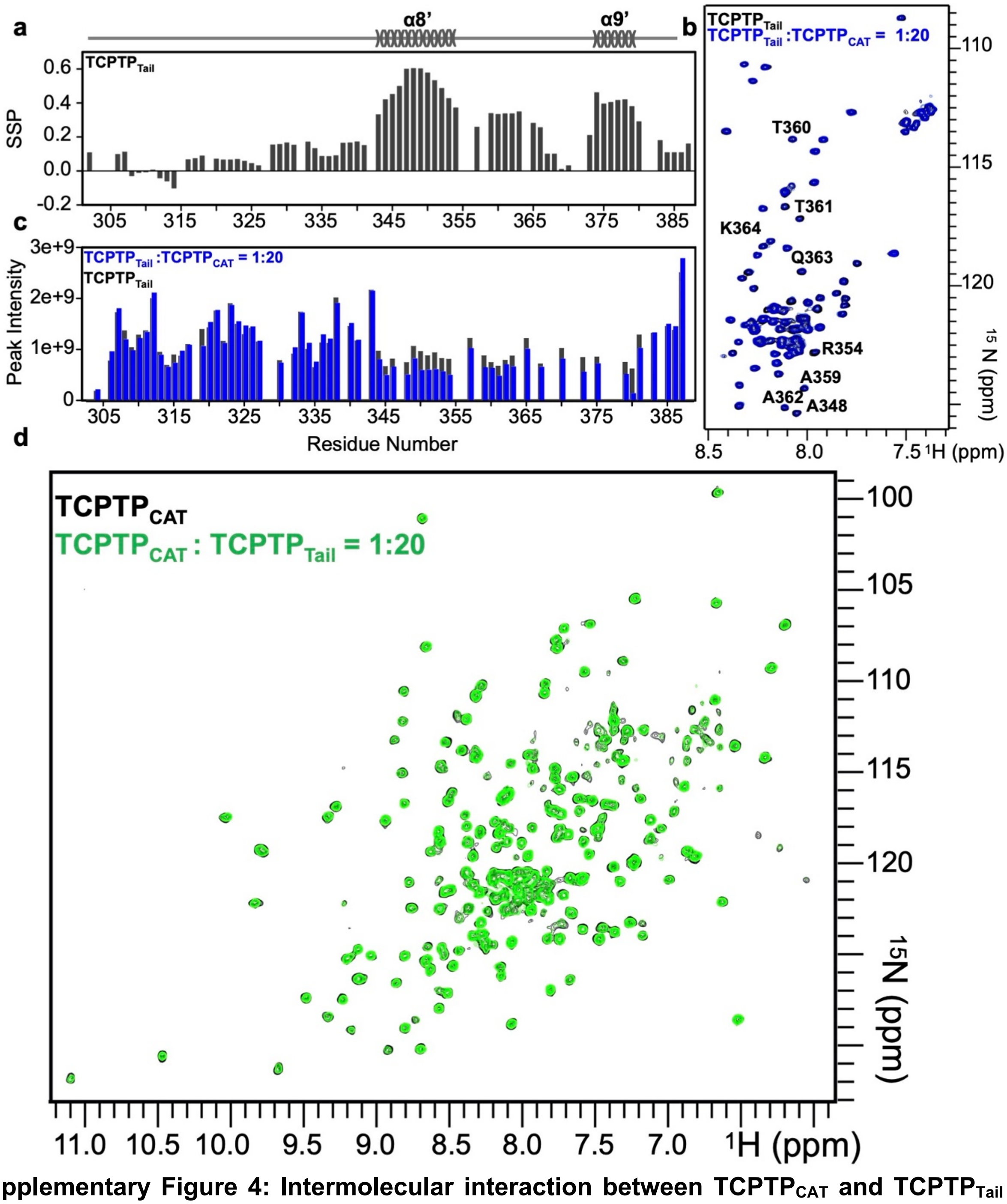

Supplementary Figure 4: Intermolecular interaction between TCPTP$_{CAT}$ and TCPTP$_{Tail}$ by NMR spectroscopy. a SSP vs TCPTP$_{Tail}$ residue numbers. Data indicate regions with transient secondary structures (SSP > 0, α helix; SSP < 0, β strand). **b** Overlay of the 2D [^{1}H,^{15}N] HQSC spectra of ^{15}N-labeled TCPTP$_{Tail}$ (black) and TCPTP$_{CAT}$-bound (1:20 ratio; navy). Residues that experience cross peaks intensity reduction upon addition of TCPTP$_{CAT}$ are annotated. **c** Cross peak intensity comparison between free TCPTP$_{Tail}$ (black) and TCPTP$_{CAT}$-bound (navy). **d** Overlay of the 2D [^{1}H,^{15}N] TROSY spectra of (^{2}H,^{15}N)-labeled TCPTP$_{CAT}$ (black) and TCPTP$_{Tail}$-bound (green).

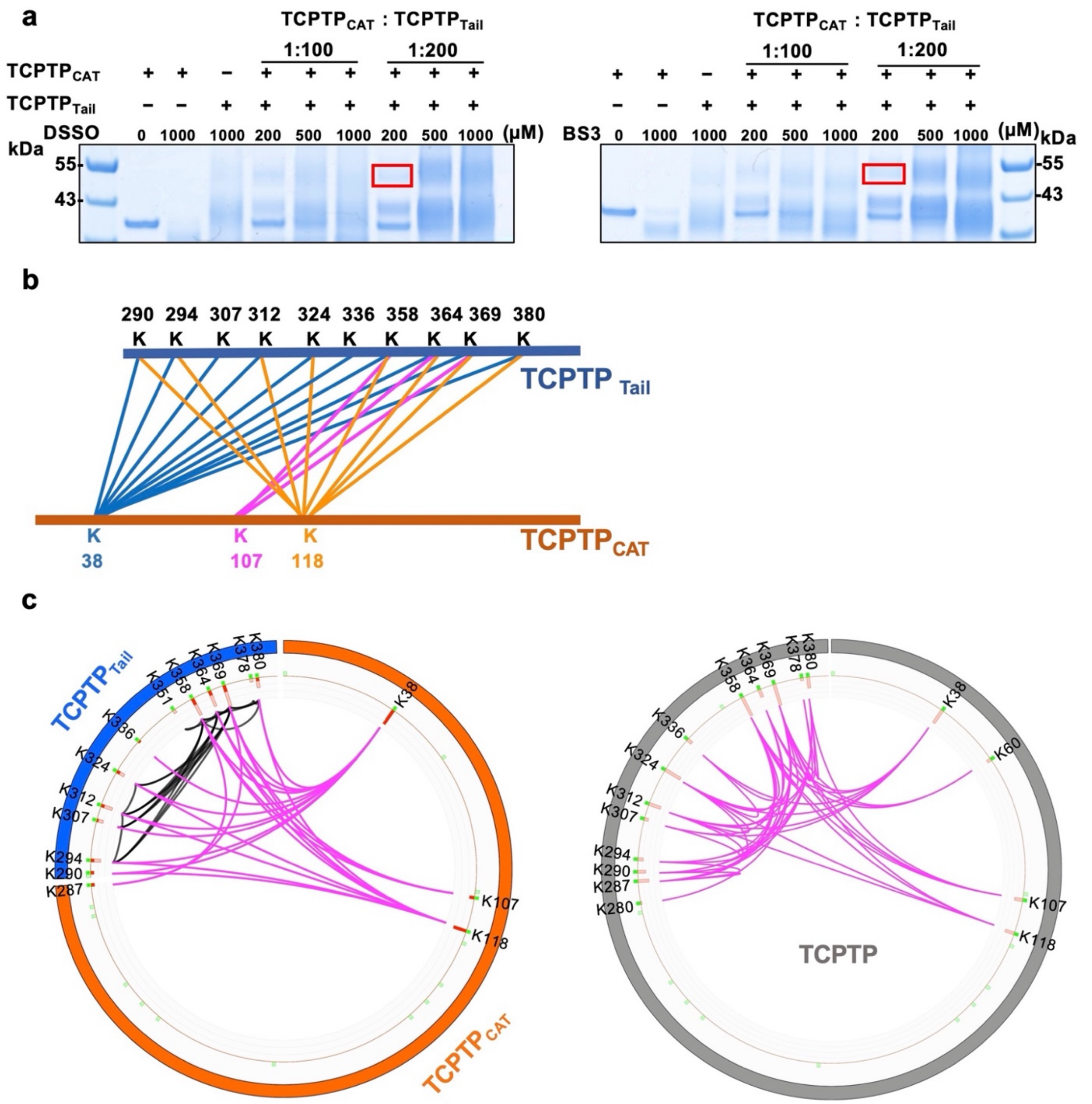

Supplementary Figure 5: Intermolecular interaction between $TCPTP_{CAT}$ and $TCPTP_{Tail}$ detected by CX-MS. a Intermolecular chemical cross-linking between $TCPTP_{CAT}$ (residues 1-288) and $TCPTP_{Tail}$ (residues 289-387) by DSSO (left; SDS-PAGE) and BS3 (right; SDS-PAGE). Each lane on SDS-PAGE is from independent reaction mixture at the indicated concentration of cross-linker, labeled on top in μM unit. The cross-linked product of complex ($TCPTP_{CAT}$ + $TCPTP_{Tail}$) appeared at the position corresponding to molecular weight of TCPTP. Red box indicates excised cross-linked product of complex from SDS-PAGE for MS analysis. **b** Cross-linking map of inter molecular interaction showing lysine residues cross-linked between $TCPTP_{CAT}$ and $TCPTP_{Tail}$. **c** Comprehensive view shows all lysine residues identified from inter- or intra-molecular crosslinking experiment by DSSO and BS3. Maps were generated by CX-Circos (left, Intermolecular CX-MS map; right, Intramolecular CX-MS map). Source data are provided as a Source Data file.

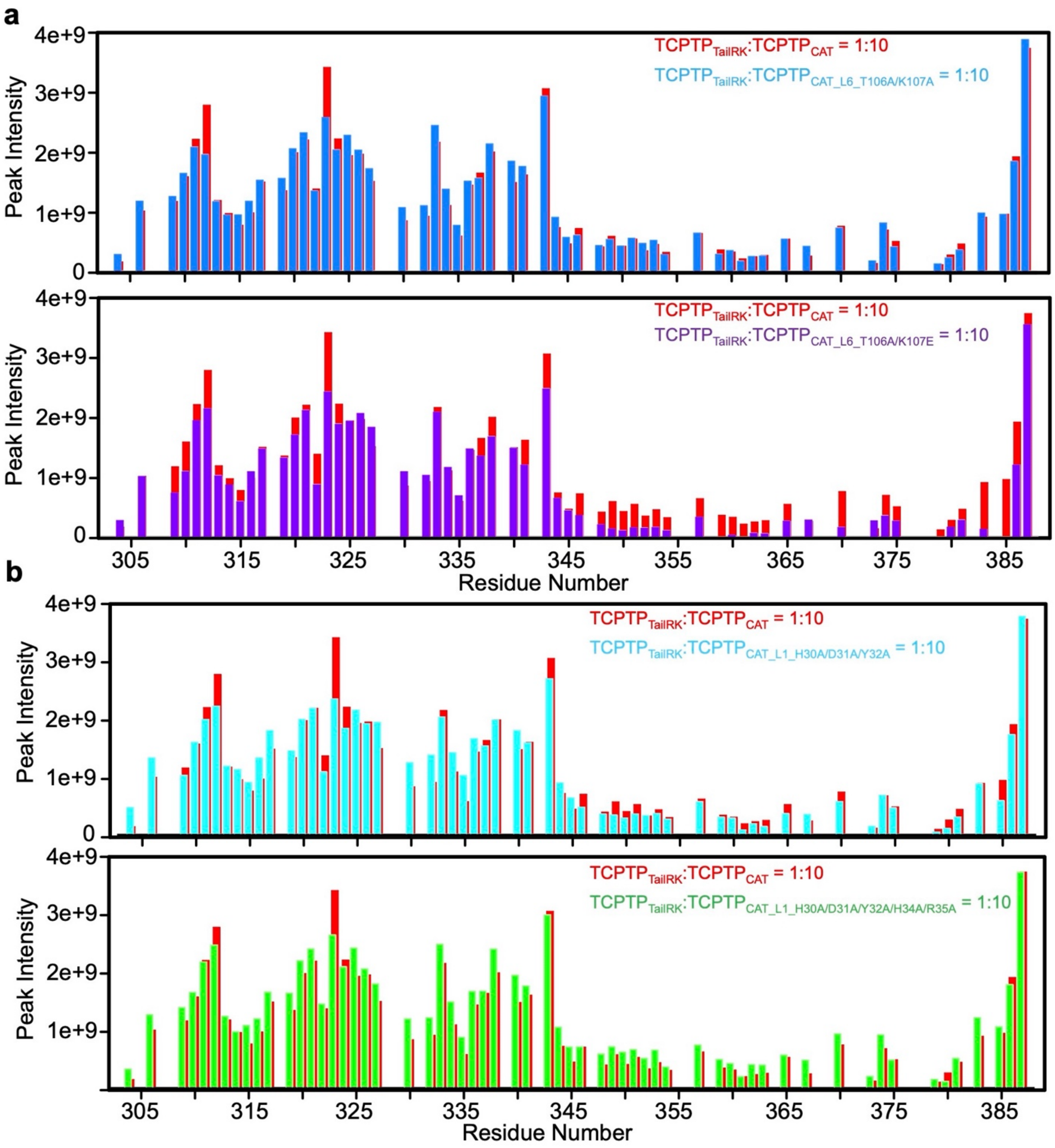

Supplementary Figure 6: 'Fuzzy' charge:charge interactions are critical for the interaction of TCPTP$_{CAT}$ and TCPTP$_{Tail}$. a Peak intensity comparison between TCPTP$_{CAT}$-bound TCPTP$_{TailRK}$ (red) and TCPTP$_{CAT_L6_T106A/K107A}$-bound TCPTP$_{TailRK}$ (blue); no difference. Peak intensity comparison between TCPTP$_{CAT}$-bound TCPTP$_{TailRK}$ (red) and TCPTP$_{CAT_L6_T106A/K107E}$-bound TCPTP$_{TailRK}$ (purple); charge reversal of K107E leads to expected stronger binding, highlighting the importance of charge in L6. **b** Peak intensity comparison between TCPTP$_{CAT}$-bound TCPTP$_{TailRK}$ (red) and TCPTP$_{CAT_L1_H30A/D31A/Y32A}$-bound TCPTP$_{TailRK}$ (turquoise); minor differences. Peak intensity comparison between TCPTP$_{CAT}$-bound TCPTP$_{TailRK}$ (red) and TCPTP$_{CAT_L1_H30A/D31A/Y32A/H34A/R35A}$-bound TCPTP$_{TailRK}$ (green); deletion of charge H34A/R35A leads to an expected weakening of binding, highlighting the importance of charge in L1.

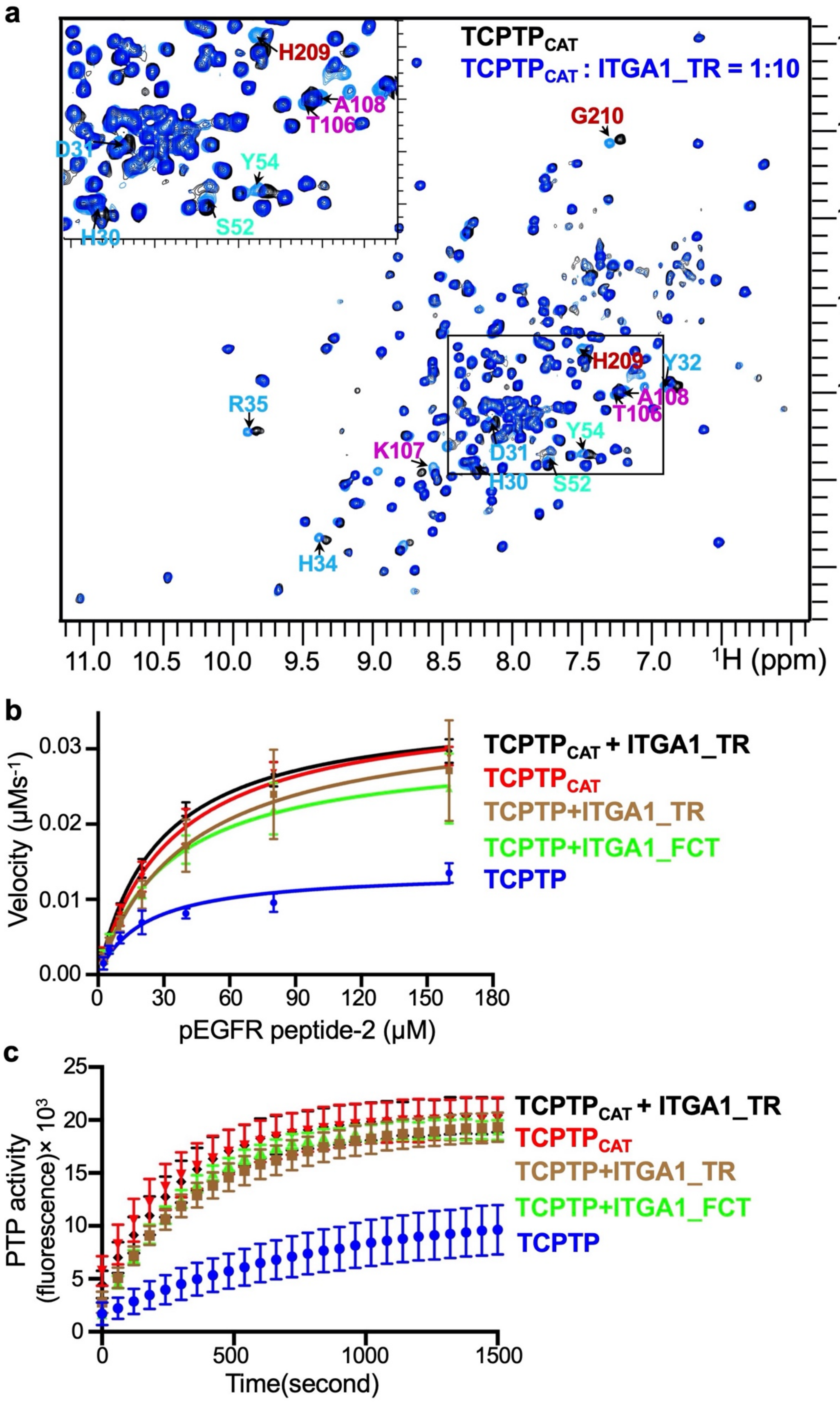
a
TCPTP$_{CAT}$
TCPTP$_{CAT}$: ITGA1_TR = 1:10
H209
A108
T106
D31
Y54
S52
H30
G210
Y32
R35
K107
H34
^{15}N (ppm)
^{1}H (ppm)
b
Velocity (µMs^{-1})
pEGFR peptide-2 (µM)
TCPTP$_{CAT}$ + ITGA1_TR
TCPTP$_{CAT}$
TCPTP+ITGA1_TR
TCPTP+ITGA1_FCT
TCPTP
c
PTP activity (fluorescence)× 10^{3}
Time(second)
TCPTP$_{CAT}$ + ITGA1_TR
TCPTP$_{CAT}$
TCPTP+ITGA1_TR
TCPTP+ITGA1_FCT
TCPTP

Supplementary Figure 7: Molecular basis for ITGA1 mediated activation of TCPTP activity. **a** Overlay of the 2D [^{1}H,^{15}N] TROSY spectra of (^{2}H,^{15}N)-labeled TCPTP$_{CAT}$ (black) and in complex with Integrin α1 peptide ITGA1_TR (navy). Residues with CSPs upon ITGA1_TR addition are annotated. Residues that changed upon IGTA1 interaction are located in the TCPTP loop L1 (green), L2 (navy), L6 (purple) and L14 (orange). **b** Catalytic activity of TCPTP and TCPTP$_{CAT}$ (residue: 1-314) in the presence or absence of synthetic ITGA1 (ITGA1_FCT or ITGA1_TR) peptides at saturation against pEGFR peptide-2, derived from the EGFR's kinase domain activation loop. **c** Catalytic activity of TCPTP and TCPTP$_{CAT}$ (residue: 1-314) in the presence or absence of synthetic ITGA1(ITGA1_FCT or ITGA1_TR) peptides at saturation against DiFMUP. Data shown in (**b**) and (**c**) are presented as the mean of nine independent reactions (*n* = *9*; mean ± SE). Source data are provided as a Source Data file.

a

```
ITGA1    KIGFFKRPLKKKMEK----------------------------------------------------------------
ITGA2    KLGFFKRKYEKMTKNPDEIDETTE--LSS--------------------------------------------------
ITGA10   KLGFFAHKKIPEEEKREE---KLE--Q----------------------------------------------------
ITGA11   KLGFFRSARRRREPGLDPTPKVLE-------------------------------------------------------
ITGAE    KCGFFKRKYQQLNLESIRKAQLKS-EN---LLEEEN-------------------------------------------
ITGAL    KVGFFKRNLKEKMEAGRGVPNGIPAEDSEQLASGQEAGD-------------PGCLKPLHEKDSESGGGKD--------
ITGAM    KLGFFKRQYKDMMSEGGPPGAEPQ-------------------------------------------------------
ITGAD    KLGFFKRHYKEMLEDKPEDTATFSGDDF-------------------------SCVAPNVPLS----------------
ITGAX    KVGFFKRQYKEMMEEANGQIAPENGTQT-------------------------PS----PPSEK---------------
ITGA3    KCGFFKRARTRAL----YEAKRQKAEMKSQPSETERLTDD-----Y---------------------------------
ITGA6    KCGFFKRSRY-DDSVPRYHAVRIRKEEREIKD--EKYIDNLEKKQWITKWNENESYS----------------------
ITGA7    KMGFFKRAKHPEATVPQYHAVKIPREDRQQFK--EEKTGTILRNNWGSPRREGPDAHPILAADGHPELGPDGHPGPGTA
ITGA4    KAGFFKRQYKSILQE----------ENR------RDSWS------YINSKSNDD-------------------------
ITGA9    KMGFFRRRYKEIIEA----------EKNRKEN--EDSWD------WVQKNQ----------------------------
ITGA2B   KVGFFKRNRPPLEED----------DEEGE-------------------------------------------------
ITGAV    RMGFFKRVRPPQEEQ----------EREQ-LQ--PHENG------EGNSET----------------------------
ITGA5    KLGFFKRSLPYGTAM----------EKAQ-LK--PPATS------DA--------------------------------
ITGA8    KCGFFDRARPPQEDM----------TDREQLT--NDKTP------EA--------------------------------
```

b

ITGA1 : KIGFFKRPLKKKMEK
ITGA10 : KLGFFAHKKIPEEEKREEKLEQ
ITGA11 : KLGFFRSARRRREPGLDPTPKVLE

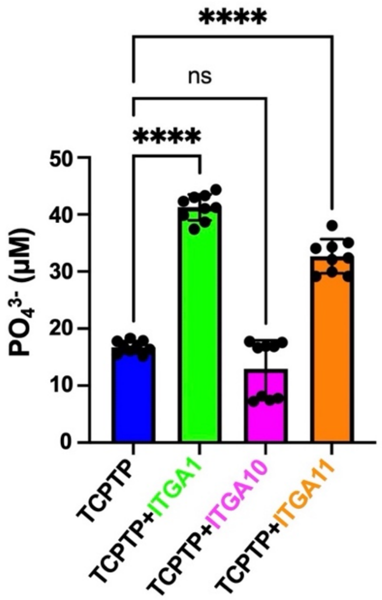

Supplementary Figure 8: TCPTP is distinctively activated by Integrin cytoplasmic tail with positive charge residue clusters. a Sequence alignment of the cytoplasmic tail for the human Integrin family. Positive charge residues are highlighted in blue, while negative charge residues are red; a black box highlights large clusters of positive charge residue. **b** Effect of ITGA1, ITGA10 & ITGA11 on the catalytic activity of TCPTP, measured against pEGFR peptide-2, derived from the EGFR's kinase domain activation loop. Data are presented as the mean of nine independent reactions (*n* = *9*; mean ± SD). **** indicates *p*-value of <0.0001, obtained from one-way ANOVA analysis with Tukey's multiple comparison test, 'ns' indicates non-significant (*p*-value = 0.0743). Peptides derived from ITGA1, ITGA10 and ITGA11 used in the assays are shown. Source data are provided as a Source Data file.

Supplementary Table 1: SAXS data collection and scattering derived parameter for TCPTP and $TCPTP_{CAT}$. Source data are provided as a Source Data file.

	TCPTP	$TCPTP_{CAT}$
**Sample details**		
Organism	*Homo sapience*	*Homo sapience*
Source	*E. coli* expressed	*E. coli* expressed
Sample Injection volume (µl)	100	100
Sample concentration ($mg.ml^{-1}$)	~15	~15
Flow rate ($ml.min^{-1}$)	0.035	0.035
**Data collection parameter**		
Beamline	BL23A1, NSRRC Taiwan	BL23A1, NSRRC Taiwan
Detector	Pilatus 1M-F	Pilatus 1M-F
Sample to detector distance (mm)	2524	2561
Column for SEC-SAXS	GE Healthcare Superdex 200 Increase 5/150 GL	Agilent Bio SEC-3, 300 Å, 4.6 X 300 mm
Wavelength (Å)	0.8266	0.8266
q range ($Å^{-1}$)	0.0005 - 0.44	0.0005 - 0.43
Absolute scaling method	Comparison with scattering from pure H_2O	Comparison with scattering from pure H_2O
Exposure time per frame (sec)	20	20
Temperature (K)	288	288
**Structural parameter**		
$I_{(0)}$ (cm^{-1}) (from Guinier)	$0.16 \pm 4.3 \times 10^{-4}$	$0.18 \pm 2.1 \times 10^{-4}$
Rg (Å) (from Guinier)	37.3 ± 0.1	27.2 ± 0.1
$I_{(0)}$ (cm^{-1}) [from $P_{(r)}$]	$0.15 \pm 1.9 \times 10^{-3}$	$0.17 \pm 1.6 \times 10^{-3}$
Rg (Å) [from $P_{(r)}$]	38.5 ± 0.9	26.6 ± 0.4
D_{max} (Å)	165	105
Porod Volume ($Å^3$)	98700	69900
Volume of correlation (V_c)	475	364
Molecular mass M_r from (V_c) (Da)	49176	39576
Calculated M_r from sequence (Da)	45440	36881
**Software employed**		
Primary data reduction	NSRRC 23A SWAXS data reduction package	NSRRC 23A SWAXS data reduction package
Data processing	PRIMUS, GNOM, ScÅtter	PRIMUS, GNOM, ScÅtter
Homology modeling	Phyre2	N. A.
Ensemble conformation modeling	MultiFoXS	N. A.
Three-dimensional graphic representation	PyMOL	N. A.

Supplementary Table 2: Enzymatic activity assay (Michaelis-Menten analysis); substrate pEGFR peptide-2, derived from the EGFR's kinase domain activation loop. Calculated kinetic parameter are presented as the mean of nine independent reactions (*n=9*; mean ± SE). Source data are provided as a Source Data file.

	k_{cat} (s^{-1})	K_m (μM)	k_{cat}/K_m ($\mu M^{-1}s^{-1}$)
**TCPTP**			
$TCPTP_{CAT}$	36.3 ± 0.9	34.0 ± 2.2	1.1 ± 0.1
TCPTP	13.8 ± 0.5	21.2 ± 2.6	0.6 ± 0.1
**ITGA1 (saturated)**			
$TCPTP_{CAT}$ + ITGA1_TR	35.6 ± 0.6	28.3 ± 1.3	1.2 ± 0.1
TCPTP + ITGA1_FCT	30.2 ± 1.2	32.8 ± 3.7	0.9 ± 0.1
TCPTP + ITGA1_TR	34.7 ± 2.3	40.9 ± 6.8	0.8 ± 0.1

Supplementary Table 3: TCPTP cross-linking analysis (intramolecular): cross-linked residues by DSSO or BS3 cross-linkers. Source data are provided as a Source Data file.

Sample	Position A Lysine Residue in TCPTP	Position B Lysine Residue in TCPTP	Score (XLinkX)
TCPTP-DSSO Cross-linker			
TCPTP-DSSO-Band	38	312	142.9
TCPTP-DSSO-Band	38	324	290.5
TCPTP-DSSO-Band	38	336	68.5
TCPTP-DSSO-Band	38	358	41.1
TCPTP-DSSO-Band	107	364	81.9
TCPTP-DSSO-Band	107	369	141.2
TCPTP-DSSO-Band	118	312	154.9
TCPTP-DSSO-Band	118	324	211.6
TCPTP-DSSO-Band	118	358	216.1
TCPTP-DSSO-Band	118	369	87.3
TCPTP-DSSO-Band	280	369	107.7
TCPTP-DSSO-Band	287	294	34.8
TCPTP-DSSO-Band	287	364	17.3
TCPTP-DSSO-Band	287	290	117.8
TCPTP-DSSO-Band	290	369	21.0
TCPTP-DSSO-Band	312	324	214.8
TCPTP-DSSO-Band	312	369	137.0
TCPTP-DSSO-Band	324	369	136.9
TCPTP-DSSO-Band	358	369	97.9
TCPTP-BS3 Cross-linker			
TCPTP-BS3-Band	38	307	81.3
TCPTP-BS3-Band	38	312	77.9
TCPTP-BS3-Band	38	324	101.7
TCPTP-BS3-Band	38	336	72.0
TCPTP-BS3-Band	38	358	44.8
TCPTP-BS3-Band	38	364	50.9
TCPTP-BS3-Band	38	369	113.9
TCPTP-BS3-Band	60	358	67.7
TCPTP-BS3-Band	60	364	41.9
TCPTP-BS3-Band	60	369	44.1
TCPTP-BS3-Band	107	358	78.0
TCPTP-BS3-Band	107	364	51.7
TCPTP-BS3-Band	107	369	113.9
TCPTP-BS3-Band	107	380	42.8
TCPTP-BS3-Band	118	324	101.7
TCPTP-BS3-Band	118	358	93.6
TCPTP-BS3-Band	118	369	50.3
TCPTP-BS3-Band	287	290	49.5
TCPTP-BS3-Band	287	358	110.4
TCPTP-BS3-Band	287	369	56.1
TCPTP-BS3-Band	287	380	52.8
TCPTP-BS3-Band	290	307	41.9
TCPTP-BS3-Band	290	358	87.7
TCPTP-BS3-Band	290	380	41.0
TCPTP-BS3-Band	294	312	42.3
TCPTP-BS3-Band	294	358	67.7

TCPTP-BS3-Band	294	380	42.6
TCPTP-BS3-Band	307	358	52.5
TCPTP-BS3-Band	312	358	85.0
TCPTP-BS3-Band	312	380	42.8
TCPTP-BS3-Band	324	312	101.5
TCPTP-BS3-Band	324	336	80.7
TCPTP-BS3-Band	324	358	104.6
TCPTP-BS3-Band	324	364	43.2
TCPTP-BS3-Band	324	369	70.4
TCPTP-BS3-Band	324	380	58.9
TCPTP-BS3-Band	336	358	67.3
TCPTP-BS3-Band	358	369	100.9
TCPTP-BS3-Band	369	380	55.3
TCPTP-BS3-Band	378	369	53.4
TCPTP-BS3-Band	378	380	55.3

Supplementary Table 4: Intermolecular cross-linking analysis between TCPTP$_{CAT}$ and TCPTP$_{Tail}$: cross-linked residue by either DSSO or BS3 cross-linker. Source data are provided as a Source Data file.

Sample	Position A Lysine Residue in TCPTP	Position B Lysine Residue in TCPTP	Score (XLinkX)
TCPTP$_{CAT}$ +TCPTP$_{Tail}$-DSSO Cross-linker			
TCPTP$_{CAT}$ +TCPTP$_{Tail}$-DSSO_Band	38	312	163.3
TCPTP$_{CAT}$ +TCPTP$_{Tail}$-DSSO_Band	38	358	129.5
TCPTP$_{CAT}$ +TCPTP$_{Tail}$-DSSO_Band	38	380	125.5
TCPTP$_{CAT}$ +TCPTP$_{Tail}$-DSSO_Band	107	358	129.4
TCPTP$_{CAT}$ +TCPTP$_{Tail}$-DSSO_Band	107	364	239.6
TCPTP$_{CAT}$ +TCPTP$_{Tail}$-DSSO_Band	107	369	194.2
TCPTP$_{CAT}$ +TCPTP$_{Tail}$-DSSO_Band	118	290	175.1
TCPTP$_{CAT}$ +TCPTP$_{Tail}$-DSSO_Band	118	294	201.2
TCPTP$_{CAT}$ +TCPTP$_{Tail}$-DSSO_Band	118	312	243.3
TCPTP$_{CAT}$ +TCPTP$_{Tail}$-DSSO_Band	118	324	259.7
TCPTP$_{CAT}$ +TCPTP$_{Tail}$-DSSO_Band	118	358	194.0
TCPTP$_{CAT}$ +TCPTP$_{Tail}$-DSSO_Band	118	364	305.1
TCPTP$_{CAT}$ +TCPTP$_{Tail}$-DSSO_Band	118	369	154.7
TCPTP$_{CAT}$ +TCPTP$_{Tail}$-DSSO_Band	118	380	164.7
TCPTP$_{CAT}$ +TCPTP$_{Tail}$-DSSO_Band	287	358	139.6
TCPTP$_{CAT}$ +TCPTP$_{Tail}$-DSSO_Band	287	369	122.5
TCPTP$_{CAT}$ +TCPTP$_{Tail}$-DSSO_Band	312	369	154.9
TCPTP$_{CAT}$ +TCPTP$_{Tail}$-DSSO_Band	324	364	150.2
TCPTP$_{CAT}$ +TCPTP$_{Tail}$-DSSO_Band	358	369	183.9
TCPTP$_{CAT}$ +TCPTP$_{Tail}$-DSSO_Band	358	380	227.4
TCPTP$_{CAT}$ +TCPTP$_{Tail}$-DSSO_Band	364	369	171.8
TCPTP$_{CAT}$ +TCPTP$_{Tail}$-DSSO_Band	364	380	245.5
TCPTP$_{CAT}$ +TCPTP$_{Tail}$-DSSO_Band	369	380	142.0
TCPTP$_{CAT}$ +TCPTP$_{Tail}$-BS3 Cross-linker			
TCPTP$_{CAT}$+TCPTP$_{Tail}$-BS3_Band	38	290	84.6
TCPTP$_{CAT}$+TCPTP$_{Tail}$-BS3_Band	38	294	81.6
TCPTP$_{CAT}$+TCPTP$_{Tail}$-BS3_Band	38	307	119.2
TCPTP$_{CAT}$+TCPTP$_{Tail}$-BS3_Band	38	312	101.7
TCPTP$_{CAT}$+TCPTP$_{Tail}$-BS3_Band	38	324	118.1
TCPTP$_{CAT}$+TCPTP$_{Tail}$-BS3_Band	38	336	77.9
TCPTP$_{CAT}$+TCPTP$_{Tail}$-BS3_Band	38	358	93.0
TCPTP$_{CAT}$+TCPTP$_{Tail}$-BS3_Band	38	364	71.7
TCPTP$_{CAT}$+TCPTP$_{Tail}$-BS3_Band	38	369	124.3
TCPTP$_{CAT}$+TCPTP$_{Tail}$-BS3_Band	38	380	52.8
TCPTP$_{CAT}$+TCPTP$_{Tail}$-BS3_Band	107	358	44.8
TCPTP$_{CAT}$+TCPTP$_{Tail}$-BS3_Band	107	369	72.0
TCPTP$_{CAT}$+TCPTP$_{Tail}$-BS3_Band	118	294	49.3
TCPTP$_{CAT}$+TCPTP$_{Tail}$-BS3_Band	118	358	78.0
TCPTP$_{CAT}$+TCPTP$_{Tail}$-BS3_Band	294	312	72.2
TCPTP$_{CAT}$+TCPTP$_{Tail}$-BS3_Band	294	358	72.0
TCPTP$_{CAT}$+TCPTP$_{Tail}$-BS3_Band	294	364	67.7
TCPTP$_{CAT}$+TCPTP$_{Tail}$-BS3_Band	294	369	72.3
TCPTP$_{CAT}$+TCPTP$_{Tail}$-BS3_Band	307	358	93.6
TCPTP$_{CAT}$+TCPTP$_{Tail}$-BS3_Band	307	364	56.2
TCPTP$_{CAT}$+TCPTP$_{Tail}$-BS3_Band	307	369	78.0

$TCPTP_{CAT}$+$TCPTP_{Tail}$-BS3_Band	312	324	40.4
$TCPTP_{CAT}$+$TCPTP_{Tail}$-BS3_Band	312	358	107.5
$TCPTP_{CAT}$+$TCPTP_{Tail}$-BS3_Band	312	364	58.6
$TCPTP_{CAT}$+$TCPTP_{Tail}$-BS3_Band	312	369	83.7
$TCPTP_{CAT}$+$TCPTP_{Tail}$-BS3_Band	324	369	72.0
$TCPTP_{CAT}$+$TCPTP_{Tail}$-BS3_Band	351	358	84.1
$TCPTP_{CAT}$+$TCPTP_{Tail}$-BS3_Band	351	369	64.4
$TCPTP_{CAT}$+$TCPTP_{Tail}$-BS3_Band	358	364	125.4
$TCPTP_{CAT}$+$TCPTP_{Tail}$-BS3_Band	358	369	106.6
$TCPTP_{CAT}$+$TCPTP_{Tail}$-BS3_Band	364	369	101.6
$TCPTP_{CAT}$+$TCPTP_{Tail}$-BS3_Band	369	378	47.8
$TCPTP_{CAT}$+$TCPTP_{Tail}$-BS3_Band	369	380	41.0
$TCPTP_{CAT}$+$TCPTP_{Tail}$-BS3_Band	378	380	77.1

Supplementary Table 5: Primers and recombinant DNA

TCPTP construct variants	***Primer sequence (5'→3')***		**SOURCE**
Mutation Primer			
$TCPTP_{TailRK}$	Forward-1	5'-GCAGATGAAACAGAGGCTAAATGAGAGGAAACGAAAAAGAAAAAGGCCAAGATTGACA-3'	This paper
	Reverse-1	5'-TGTCAATCTTGGCCTTTTTCTTTTTCGTTTCCTCTCATTTAGCCTCTGTTTCATCTGC-3'	This paper
	Forward-2	5'-GCAGATGAAACAGAGGCTAAGGAAAAGGAAACGAAAAAGAAAAAGGCCAAGATTGACA-3'	This paper
	Reverse-2	5'-TGTCAATCTTGGCCTTTTTCTTTTTCGTTTCCTTTTCCTTAGCCTCTGTTTCATCTGC-3'	This paper
TCPTP_C-terminal mutant	Forward	5' **(P)-**GGCGGCGCCAAGATTGACAGACACC-3'	This paper
	Reverse	5' **(P)-**GCCGCCGCTTCATTCTCATTTAGCCTC -3'	This paper
TCPTP_L1 loop mutant	Forward	5' **(P)-**CCGGCGGCGGTGGCCAAGTTTCCAGAA - 3'	This paper
	Reverse	5' **(P)-**CGCCGCCGCGGACTCATTTCGAATTTCC -3'	This paper
TCPTP_L1_loop_H30A/D31D/Y32A mutant	Forward	5'-TGTACTTGGAAATTCGAAATGAGTCCGCTGCCGCTCCTCATAGAGTGGCCAAGTTTCCAG-3'	This paper
	Reverse	5'-CTGGAAACTTGGCCACTCTATGAGGAGCGGCAGCGGACTCATTTCGAATTTCCAAGTACA-3'	This paper
TCPTP_L6 loop_T106A/K107A mutant	Forward	5'-TTATGGTTTGGCAGCAGAAGGCCGCAGCAGTTGTCATGCTGAACC-3'	This paper
	Reverse	5'-GGTTCAGCATGACAACTGCTGCGGCCTTCTGCTGCCAAACCATAA-3'	This paper
TCPTP_L6 loop_T106A/K107E mutant	Forward	5'-TATGGTTTGGCAGCAGAAGGCCGAGGCAGTTGTCATGCTGAACC-3'	This paper
	Reverse	5'-GGTTCAGCATGACAACTGCCTCGGCCTTCTGCTGCCAAACCATA-3'	This paper
Cloning Primer			
TCPTP (1-387)-pMCSG7 Vector	Forward	5'-TACTTCCAATCCAATGCGATGCCCACCACCATCGAG -3'	This paper
	Reverse	5'-TTATCCACTTCCAATGTTATTAGGTGTCTGTCAATCTTGG -3'	This paper
$TCPTP_{CAT}$ (1-314)-pMCSG7 Vector	Reverse	5'-TTATCCACTTCCAATGTTATTAATTGTATTTTTCAGTCATT -3'	This paper

TCPTP$_{CAT}$ (1-288)-pMCSG7 Vector	Reverse	5’-TTATCCACTTCCAATGTTATTATCGTTTCTGTATACTAGAATC -3’	This paper
TCPTP$_{Tail}$ (289-387)-pMCSG7 Vector	Forward	5’-TACTTCCAATCCAATGCGTGGAAAGAACTTTCTAAGGAAG -3’	This paper
TCPTP (1-387)-pRP1B Vector	Forward	5’- GAATTTCGGATCCATGCCCACCACCATC -3’	This paper
	Reverse	5’- GAAATTCCCTCGAGTTAGGTGTCTGTCAATC -3’	This paper
TCPTP$_{CAT}$ (1-302)-pRP1B Vector	Forward	5’-GGAAGACTTATCTCCTGCCTTTGATTAGTCACCAAACAAAATAATGACT -3’	This paper
	Reverse	5’-AGTCATTATTTTGTTTGGTGACTAATCAAAGGCAGGAGATAAGTCTTCC -3’	This paper
TCPTP$_{Tail}$ (303-387)-pRP1B Vector	Forward	5’-ATTCGCGGATCCCATTCACCAAACAAAATAATGACT -3’	This paper
	Reverse	5’- GTGGTGCTCGAGTTAGGTGTCTGTCAATC -3’	This paper

Note: 5’**(P)-** indicate phosphorylated primer at 5’ end.
