# Supplementary material for: The catalytic activity of TCPTP is auto-regulated by its intrinsically disordered tail and activated by Integrin alpha-1
Source: Nat Commun. 2022 Jan 10;13:94. doi: 10.1038/s41467-021-27633-6 (PMC8748766; doi:10.1038/s41467-021-27633-6)
Supplement: Supplementary file 3 — Reporting Summary [file 41467_2021_27633_MOESM3_ESM.pdf]

nature research

Corresponding author(s): Wolfgang Peti & Tzu-Ching Meng

Last updated by author(s): Nov 27, 2021

Reporting Summary

Nature Research wishes to improve the reproducibility of the work that we publish. This form provides structure for consistency and transparency in reporting. For further information on Nature Research policies, see our Editorial Policies and the Editorial Policy Checklist.

Statistics

For all statistical analyses, confirm that the following items are present in the figure legend, table legend, main text, or Methods section.

n/a	Confirmed	
☐	☒	The exact sample size (*n*) for each experimental group/condition, given as a discrete number and unit of measurement
☐	☒	A statement on whether measurements were taken from distinct samples or whether the same sample was measured repeatedly
☐	☒	The statistical test(s) used AND whether they are one- or two-sided<br>*Only common tests should be described solely by name; describe more complex techniques in the Methods section.*
☒	☐	A description of all covariates tested
☐	☒	A description of any assumptions or corrections, such as tests of normality and adjustment for multiple comparisons
☐	☒	A full description of the statistical parameters including central tendency (e.g. means) or other basic estimates (e.g. regression coefficient) AND variation (e.g. standard deviation) or associated estimates of uncertainty (e.g. confidence intervals)
☐	☒	For null hypothesis testing, the test statistic (e.g. *F*, *t*, *r*) with confidence intervals, effect sizes, degrees of freedom and *P* value noted<br>*Give P values as exact values whenever suitable.*
☒	☐	For Bayesian analysis, information on the choice of priors and Markov chain Monte Carlo settings
☒	☐	For hierarchical and complex designs, identification of the appropriate level for tests and full reporting of outcomes
☒	☐	Estimates of effect sizes (e.g. Cohen's *d*, Pearson's *r*), indicating how they were calculated

Our web collection on statistics for biologists contains articles on many of the points above.

Software and code

Policy information about availability of computer code

Data collection	Tecan i-control 2.0.10.0, TopSpin 4.0.5
Data analysis	GraphPad Prism-9.0, CARA, CcPNMR, PRIMUS and GNOM(ATSAS suit- 2.8.3), ScÅtter3, PD-XLinkX, Proteome DiscovererTM 2.4, 23A SWAXS data reduction package(NSRRC, Taiwan)

For manuscripts utilizing custom algorithms or software that are central to the research but not yet described in published literature, software must be made available to editors and reviewers. We strongly encourage code deposition in a community repository (e.g. GitHub). See the Nature Research guidelines for submitting code & software for further information.

Data

Policy information about availability of data

All manuscripts must include a data availability statement. This statement should provide the following information, where applicable:

- Accession codes, unique identifiers, or web links for publicly available datasets
- A list of figures that have associated raw data
- A description of any restrictions on data availability

All NMR chemical shifts have been deposited in the BioMagResBank with entry ID BMRB 50903 [https://dx.doi.org/10.13018/BMR50903], 50904 [https://dx.doi.org/10.13018/BMR50904] and 50905 [https://dx.doi.org/10.13018/BMR50905]. Chemical cross-linking mass spectrometry proteomics data have been deposited to the ProteomeXchange Consortium via the PRIDE partner repository with the dataset identifier PXD029256 [https://www.ebi.ac.uk/pride/archive/projects/PXD029256]. Two crystal structures published previously (PDB ID: 1L8K [https://www.rcsb.org/structure/1L8K] and PDB ID: 5K9W [https://www.rcsb.org/structure/1L8K]) were used in this study. Uncropped and unprocessed scans of the blots are provided in the Source Data file. Primers and recombinant DNA used in this study are summarized in Supplementary Table 5. The source data underlying Figures-1d, 2a-b, 4a-b, 5a, 6a, 6d, Table-1, Supplementary Figures- 2a-c, 3a-b, 5a-c, 7b-c, 8b and Supplementary Tables 1-4 are provided as a Source Data file.

Field-specific reporting

Please select the one below that is the best fit for your research. If you are not sure, read the appropriate sections before making your selection.

- [x] Life sciences
- [ ] Behavioural & social sciences
- [ ] Ecological, evolutionary & environmental sciences

For a reference copy of the document with all sections, see nature.com/documents/nr-reporting-summary-flat.pdf

Life sciences study design

All studies must disclose on these points even when the disclosure is negative.

Sample size	Sample size was chosen based on previous experience, No statistical calculation was performed to determine sample size.
Data exclusions	No data were excluded.
Replication	NMR, SAXS and mass spectrometry experiments were performed once on one sample for each condition. For enzymatic activity and kinetic analysis, the data are presented based on multiple independent replicates. Results were reproduced successfully in each run with a similar conclusion.
Randomization	This study did not require randomization. All experiments were performed in vitro under well-controlled condition.
Blinding	For the in vitro NMR, SAXS, mass spectrometry, enzymatic activity and kinetic experiments described in this manuscript, blinding was not possible as the researchers must be fully aware of the samples under analysis in order to interpret the results.

Reporting for specific materials, systems and methods

We require information from authors about some types of materials, experimental systems and methods used in many studies. Here, indicate whether each material, system or method listed is relevant to your study. If you are not sure if a list item applies to your research, read the appropriate section before selecting a response.

Materials & experimental systems

n/a	Involved in the study	
x		Antibodies
x		Eukaryotic cell lines
x		Palaeontology and archaeology
x		Animals and other organisms
x		Human research participants
x		Clinical data
x		Dual use research of concern

Methods

n/a	Involved in the study	
x		ChIP-seq
x		Flow cytometry
x		MRI-based neuroimaging
